# Mitochondrial DNA *D-loop* variants correlate with a primary open-angle glaucoma subgroup

**DOI:** 10.3389/fopht.2023.1309836

**Published:** 2024-01-17

**Authors:** Antoni Vallbona-Garcia, Patrick J. Lindsey, Rick Kamps, Alphons P. M. Stassen, Nhan Nguyen, Florence H. J. van Tienen, Ilse H. J. Hamers, Rianne Hardij, Marike W. van Gisbergen, Birke J. Benedikter, Irenaeus F. M. de Coo, Carroll A. B. Webers, Theo G. M. F. Gorgels, Hubert J. M. Smeets

**Affiliations:** ^1^ University Eye Clinic Maastricht, Maastricht University Medical Center, Maastricht, Netherlands; ^2^ Department of Toxicogenomics, Maastricht University, Maastricht, Netherlands; ^3^ School for Mental Health and Neuroscience, Maastricht University, Maastricht, Netherlands; ^4^ Department of Clinical Genetics, Maastricht University Medical Center, Maastricht, Netherlands; ^5^ Department of Dermatology, Maastricht University Medical Center, Maastricht, Netherlands; ^6^ GROW School for Oncology and Reproduction, Maastricht University, Maastricht, Netherlands

**Keywords:** mitochondria, glaucoma, mtDNA, D-loop (control region), POAG, mtDNA replication

## Abstract

**Introduction:**

Primary open-angle glaucoma (POAG) is a characteristic optic neuropathy, caused by degeneration of the optic nerve-forming neurons, the retinal ganglion cells (RGCs). High intraocular pressure (IOP) and aging have been identified as major risk factors; yet the POAG pathophysiology is not fully understood. Since RGCs have high energy requirements, mitochondrial dysfunction may put the survivability of RGCs at risk. We explored in buffy coat DNA whether mtDNA variants and their distribution throughout the mtDNA could be risk factors for POAG.

**Methods:**

The mtDNA was sequenced from age- and sex-matched study groups, being high tension glaucoma (HTG, n=71), normal tension glaucoma patients (NTG, n=33), ocular hypertensive subjects (OH, n=7), and cataract controls (without glaucoma; n=30), all without remarkable comorbidities.

**Results:**

No association was found between the number of mtDNA variants in genes encoding proteins, tRNAs, rRNAs, and in non-coding regions in the different study groups. Next, variants that controls shared with the other groups were discarded. A significantly higher number of exclusive variants was observed in the D-loop region for the HTG group (~1.23 variants/subject), in contrast to controls (~0.35 variants/subject). In the D-loop, specifically in the 7S DNA sub-region within the Hypervariable region 1 (HV1), we found that 42% of the HTG and 27% of the NTG subjects presented variants, while this was only 14% for the controls and OH subjects. As we have previously reported a reduction in mtDNA copy number in HTG, we analysed if specific D-loop variants could explain this. While the majority of glaucoma patients with the exclusive D-loop variants m.72T>C, m.16163 A>G, m.16186C>T, m.16298T>C, and m.16390G>A presented a mtDNA copy number below controls median, no significant association between these variants and low copy number was found and their possible negative role in mtDNA replication remains uncertain. Approximately 38% of the HTG patients with reduced copy number did not carry any exclusive D-loop or other mtDNA variants, which indicates that variants in nuclear-encoded mitochondrial genes, environmental factors, or aging might be involved in those cases.

**Conclusion:**

In conclusion, we found that variants in the D-loop region may be a risk factor in a subgroup of POAG, possibly by affecting mtDNA replication.

## 1 Introduction

Glaucoma comprises a group of complex optic neurodegenerative diseases, characterized by progressive degeneration of optic nerve cells, the retinal ganglion cells (RGCs) (1, 2). Glaucoma is one of the leading causes of irreversible blindness worldwide. The most common form of glaucoma is primary open-angle glaucoma (POAG) (2–4). Several risk factors influence disease onset and progression, the main ones are aging and high intraocular pressure (IOP, >21 mmHg) (5–7). High IOP is the current main therapy target, although reduction of IOP does not prevent the progression of the disease in all patients. Glaucoma also occurs without statistically increased IOP (2, 8). Variants in more than 150 genes have been linked to glaucoma and its phenotypic traits. Thus, the disease is multifactorial and heterogeneous with several genetic and non-genetic risk factors, which can be involved in onset and progression (9, 10).

The mechanism underlying the death of the RGCs in glaucoma is not fully understood. Since RGCs have a relatively high energy demand, suboptimal mitochondrial function has been suggested as one of the factors in the pathophysiology (11–16). Mitochondria produce energy through the oxidative phosphorylation system (OXPHOS). OXPHOS dysfunction leads to cellular and tissue energy deficits and a range of heterogeneous diseases called primary mitochondrial diseases (MD). MD are caused by defective or absent mitochondrial proteins, which can be caused by pathogenic variants in nuclear-encoded mitochondrial genes or in the mitochondrial DNA (mtDNA). Some MD present optic neuropathy phenotypes, displaying the importance of the energy supply to the optic nerve (16–19). As mitochondrial function also declines during aging, it has been suggested to contribute to the pathophysiology of complex neurodegenerative diseases e.g., glaucoma, Alzheimer and Parkinson (20, 21). The genetic component in those diseases is not as clear as in primary MD and other factors will likely be involved in causing a mitochondrial deficiency (22, 23). In case of glaucoma, mutations in mitochondrially-related nuclear (*OPA1, OPTN, MFN1, MFN2*) and mtDNA genes have been previously associated with an increased disease development risk (10, 24–27).

The mtDNA is a circular double-stranded DNA that encodes 13 proteins, which are essential OXPHOS subunits, 22 tRNAs and 2 rRNAs, which are crucial for the mtDNA encoded protein synthesis. Even if most proteins (>1000) are encoded by the nuclear DNA, mtDNA encoded proteins are equally essential for the production of ATP (28, 29). The mtDNA also presents a major non-coding region (NCR) between positions 16024 and 576. This region is also called the *D-loop* region as it has a special structure called *displacement loop (D-loop)*, which is important for replication and transcription of the mtDNA (30, 31), although the exact mechanism is not fully resolved yet. The mtDNA is present in multiple copies in each mitochondrion and each cell. Therefore, mtDNA mutations can appear in homoplasmy (all copies harbor the mutation) or heteroplasmy (part of the copies harbor the mutation). In the case of heteroplasmic pathogenic variants, the percentage of mtDNA mutated copies is predictive for the phenotypic outcome, based on the severity of the OXPHOS deficiency (19, 32–35). With the advances in next-generation sequencing (NGS), mutations in the mtDNA have been extensively studied in both patients and controls (36–38). Understanding these variants’ effect in multifactorial neurodegenerative diseases such as glaucoma is crucial for understanding how mtDNA-related genetics could influence the progression and physiopathology of the disease (35, 38).

Since optic nerve biopsies are virtually impossible to obtain, it is difficult to study the mitochondrial genome in RGCs and the optic nerve itself. Therefore, we performed NGS in blood mtDNA in order to investigate if mtDNA variants and its presence in certain regions could reflect or predispose to a systemic reduction in OXPHOS capacity later in life (39, 40), and therefore may constitute a genetically defined risk factor for glaucomatous degeneration. The mtDNA genome was studied in two patient groups: high tension glaucoma (HTG, POAG patients with high IOP at diagnosis), normal tension glaucoma (NTG, POAG patients without high IOP at diagnosis) (41, 42), and compared with healthy subjects with diagnosed ocular hypertension (OH) and healthy control subjects.

## 2 Methods

### 2.1 Study population

Data and material were obtained from the Eye Tissue Bank Maastricht (ETBM), which collects clinical data and biomaterial from glaucoma and cataract patients who visit the University Eye Clinic Maastricht. The population consists of subjects from the province of Limburg, in the south of the Netherlands. All patients and controls included had been diagnosed by expert ophthalmologists and had signed beforehand an informed consent that their biomaterial could be used for research purposes. The study was approved by the ethical committee of the Maastricht University Medical Center (METC 2018-0935-A-10). The following diagnostic criteria were applied to define POAG: open anterior chamber angle as observed by gonioscopy; no physical abnormalities in the anterior chamber that suggest pigment dispersion or pseudoexfoliation; glaucomatous changes of the optic nerve head and glaucomatous visual field defects on perimetry. POAG patients were included and further divided into two groups based on IOP measured at the time of diagnosis and before treatment initiation: HTG patients with an IOP > 21 mmHg and NTG patients with an IOP ≤ 21 mmHg. Control subjects were included and classified into two groups depending on the presence of elevated IOP. Subjects that presented an IOP > 21 mmHg but did not present the glaucomatous diagnosis criteria, were classified as OH healthy subjects. Subjects without glaucomatous diagnosis nor IOP > 21 mmHg, who underwent cataract surgery, were selected as controls. Exclusion criteria were the presence of heart-related disorders, blood and platelet cells related disorders, cancer, diabetes, liver-related diseases, kidney disorders (except kidney stones), lung diseases (except bronchitis), peripheral nor central nervous system related disorders, and a history of other eye disorders (uveitis, age-related macular degeneration, or diabetic retinopathy) at the moment of blood extraction. In total, 141 subjects from which 71 are HTG patients, 33 are NTG patients, 7 are OH subjects and 30 are controls were analyzed in this study. The patients and controls in this study are part of the subjects (141/175) analyzed for mtDNA copy number and 4977 base pair deletion in a previous study in our group (43). Although the OH is a small group of subjects, it is interesting to study its mtDNA due to possible linkage to phenotypic resistance to optic degeneration (44).

### 2.2 DNA isolation

Buffy coat samples were collected in the ETBM. DNA from the subjects in our study cohort was isolated at the department of Clinical Genetics at Maastricht University Medical Center. Isolations were performed on subjects’ buffy coat obtained from the centrifugation of EDTA blood (pre-cooled centrifuge at 4°C, 2000xg, 10 minutes) with the QIAsymphony DSP Midi Kit (Qiagen) and the QIAsymphony platform (Qiagen) according to manufacturer’s instructions.

### 2.3 MtDNA amplification and library preparation for NGS

Two overlapping mtDNA fragments of 8.2 kilobases (Fragment 1 from position 708 to 8916) and 8.5 kilobases (Fragment 2 from position 8825 to position 802) covering the whole mitochondrial genome (16.569 kilobases) for each study subject were produced by long-range PCR with the following set up: 30 seconds at 98°C, 32 cycles of 10 seconds at 98°C, 20 seconds at 67°C and 2 minutes 50 seconds at 72°C and, out of the cycle, a final elongation step of 10 minutes at 72°C. For each fragment, PCR reactions were performed in a final volume of 50 µl containing GC buffer 1X (Thermofisher), 260 µM dNTP mix (Bioline), 1 unit of Phusion Hot Start DNA pol II (Thermofisher), 0.5 µM of forward primers (Fragment 1 5’ *CGTTCCAGTGAGTTCACCCT* 3’; Fragment 2 5’ *TAAACCTAGCCATGGCCATC* 3’) and reverse primer (Fragment 1 Reverse primer 5’ *GGTAAGAAGTGGGCTAGGGC* 3’; Fragment 2 5’ *TGTGGCTAGGCTAAGCGTTT* 3’) and 50 ng of DNA template. In order to check the size of the fragments 5 µl of PCR product were used to perform electrophoresis on a 1% agarose gel, visualized with SYBR Safe (Invitrogen) 1:100 with the D-DiGit Gel Scanner (Li-Cor). The fragments were purified with AMPure XP beads (Beckman Coulter), pooled in equimolar amounts to 1nM. 1 ng of purified mtDNA pooled fragments was as input for the library preparation. According to manufacturer’s protocol, the Nextera XT DNA Library Preparation Kit (Illumina) and Nextera XT DNA Index Kit V2 were used to prepare indexed paired-end DNA libraries. The libraries from the different subjects were normalized to an equal molarity (1 nM) and equal volumes of normalized libraries were pooled in a single recipient. The pooled library was diluted to the recommended loading concentration (~100-200 pM) and the control library Phix V3 (2%) (Illumina) was added to the pool. 20 µl of the pooled diluted library were introduced in the iSeq100 platform cartridge with the paired-end 300 cycle Iseq100 Reagent v2 (Illumina). Cluster generation and sequencing reactions were produced on a single lane flow cell from the mentioned kit. All DNA concentration measurements were performed with the Qubit dsDNA High Sensitivity Assay Kit and the Qubit Fluorometer (Life Technologies).

### 2.4 Mitochondrial NGS pipeline

The processing of the next generation data followed a standard pipeline. First, the raw data from the Iseq100 platform was demultiplexed using bcl2fastq v2.20.0 (Illumina) and then the fastQ files were checked with fastqc v0.11.9 (45). The fastQ files were trimmed and filtered (in the following order: removed the first left 11 bases, removed bases on the right to obtain reads of length 139, removed reads with stretches of 10 or more Ns, removed bases from both the left and right side using a window of 8 bases with mean quality of 33, and removed reads if the remaining length was less than 35 bases) using prinseq v0.20.4 (46). The reads were then aligned to the mitochondrial reference (GRCh38.p13) using bwa v0.7.17 (47) and sorted with samtools v1.15.1 (48). Within GATK v4.4.0.0 (49), the duplicates were then marked (MarkDuplicates), the bam file was validated (ValidateSamFile) and fixed (FixMateInformation and SetNmMdAndUqTags) if necessary, the variants were called using Mutect2, and calls were filtered (LearnReadOrientationModel and FilterMutectCalls). Mutect2 flagged mtDNA positions were excluded from the latter variant analysis. The vcf files were annotated using Annovar v2021-02-08 (50) with additional in-house formatted databases (ensembl v38, avsnp150, dbnsfp42a, phastCons, phyloP, SIFT4G). Finally, the mtDNA haplogroups were called with Haplogrep 3.2.1 from the vcf file (Heteroplasmy level 0.95, Phylotree 17 – Forensic Updated 1.2, Kulcynski distance function) (51).

For this study, only homoplasmic variants were studied, which were defined as variants with an alternative allele with ≥95% of heteroplasmy (52, 53). A coverage depth threshold of 50 was determined using the binomial probability distribution (54) to have a percentage of false positives below 0.001% when a Q20 score (0.01%) is used as sequencing error rate.

### 2.5 Statistical analysis

The number of variants per subject were analyzed using Poisson and zero-inflated Poisson regression model including the study group (HTG, NTG, OH or control), mtDNA regions/genes, and interaction among these for both the main regression and the zero-inflated part. The relation between mtDNA copies and the number of variants per subject was checked using a lognormal regression and the same variables. The inference criterion used for comparing the models is their ability to predict the observed data, i.e., models are compared directly through their minimized minus log-likelihood. When the numbers of parameters in models differ, they are penalized by adding the number of estimated parameters, a form of the Akaike information criterion (AIC) (55). The model under consideration was found to be better suited if the AIC decreased compared to the previous model. All statistical analysis presented were performed using the freely available program R v4.3.1 (56), the ‘zeroinfl’ function (57) of the publicly available library ‘pscl’ (58) for the zero-inflated Poisson model and the ‘glm’ function from the publicly available library ‘stats’ (59) for the Poisson model.

## 3 Results

### 3.1 Study population

The characteristics of study population have been described in detail in Vallbona-Garcia et al. (43)(see methods). In summary, sex and age-matched patients were selected from study groups namely, HTG (POAG with IOP > 21 mmHg at diagnosis), NTG (POAG with IOP ≤ 21 mmHg at diagnosis), OH subjects (IOP > 21 mmHg but no glaucomatous degeneration observed), and healthy controls who only underwent cataract surgery. Whole mtDNA NGS was performed on a representative sample of around ~80% of the subjects (71/97 HTG, 33/37 NTG, 7/9 OH, 30/32 controls), from the previously mentioned study. The OH group, although comprised of healthy individuals, has been analysed in part of the study together with the patient groups HTG and NTG. This has been done to find possible associations between variants and mtDNA regions and high IOP which is present in both HTG and OH.

### 3.2 Haplogroup distribution

**Figure 1:**
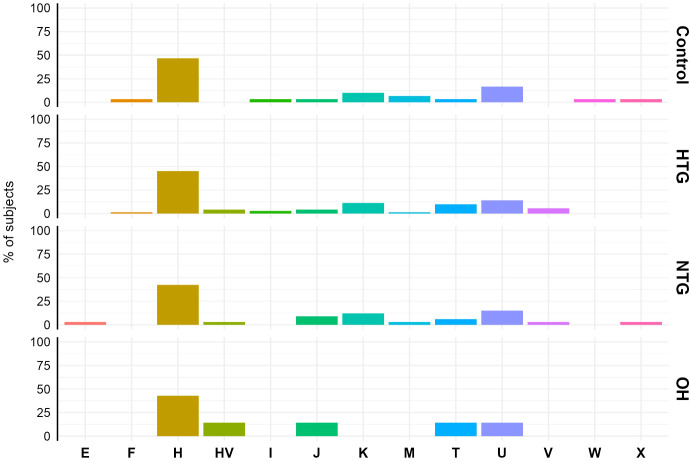
Mitochondrial haplogroup distribution in Controls, HTG, NTG and OH subjects. No difference in distribution was observed between haplogroups (displayed in the x-axis) and study groups. The data is displayed as the % of subjects pertaining to the different haplogroups in each study group. Obtained vcf files through the mutect2 tool were used in Haplogrep 3.2.1 to annotate the mitochondrial haplogroups based on the phylogenetic tree Phylotree 17 - Forensic Updated 1.2 with distance function Kulczynski. HTG, high tension glaucoma; NTG, normal tension glaucoma; OH, ocular hypertensive.

First, although the sample size of our study is too small for a proper haplogroup association study, we briefly analysed the haplogroup distribution in the study population to see if there was a major difference between the study groups. The haplogroup distribution was similar among the different groups. Haplogroup H was the predominant haplogroup, with ~40-50% of the subjects in each of the groups being part of it (**Figure 1**).

### 3.3 Analysis of the distribution of homoplasmic variants in the mtDNA

**Figure 2:**
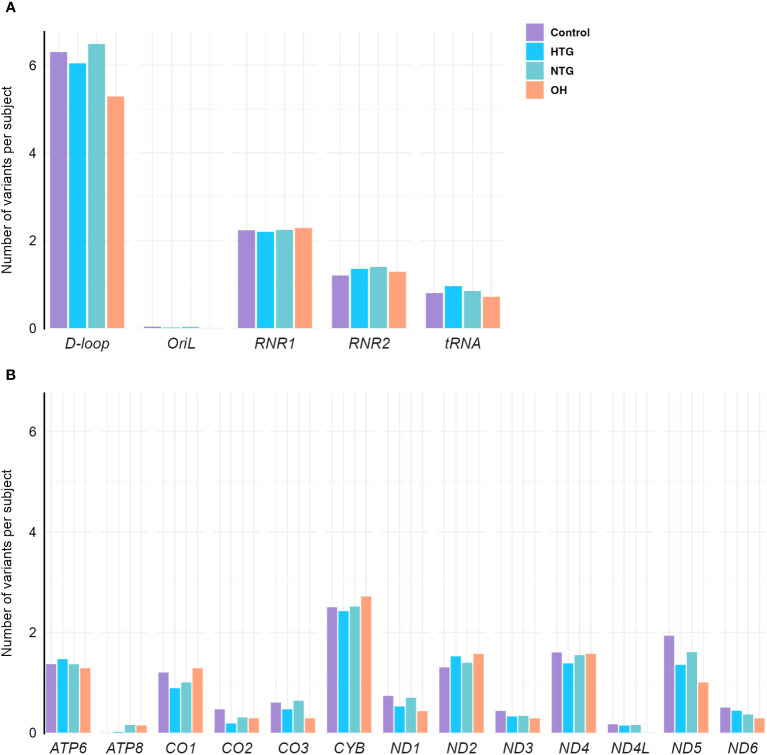
Distribution of homoplasmic variants per subject in the mtDNA **(A)** non-coding *D-loop* and *Oril* regions, *RNR1*, *RNR2* and *tRNA* coding regions **(B)** protein-coding regions of OXPHOS subunits *ATP6*, *ATP8*, *CO1*, *CO2*, *CO3*, *CYB*, *ND1*, *ND2*, *ND3*, *ND4*, *ND4L*, *ND5*, and *ND6*, in the Controls, HTG, NTG, and OH subjects. No differences between groups are observed when the number of variants in each subject per group and per region are fitted to a Poisson model. Data is displayed as the number of variants per subject. The total number of variants in each gene and group is divided by the respective size of the group. HTG, high tension glaucoma; NTG, normal tension glaucoma; OH, Ocular hypertensive.

As a next step, we explored the distribution of homoplasmic variants in different regions of the mtDNA. For that, variants were arranged in non-coding regions (*D-loop, OriL*) and protein-coding (*ND1, ND2, ND3, ND4, ND4L, ND5, ND6, CO1, CO2, CO3, CYB, ATP6, ATP8*), tRNAs (*TA, TC, TD, TE, TF, TG, TH, TI, TK, TL1, TL2, TM, TN, TP, TQ, TR, TS1, TS2, TT, TV, TW, TY*) and rRNA genes (*RNR1, RNR2*). The number of homoplasmic variants per subject and per mtDNA region of all 4 groups was fitted to a Poisson model, and the best-fitting model based on lower AIC was selected (see methods). No differences were found between the groups in any of the non-coding (**Figure 2A**) or coding regions (**Figure 2B**).

**Figure 3:**
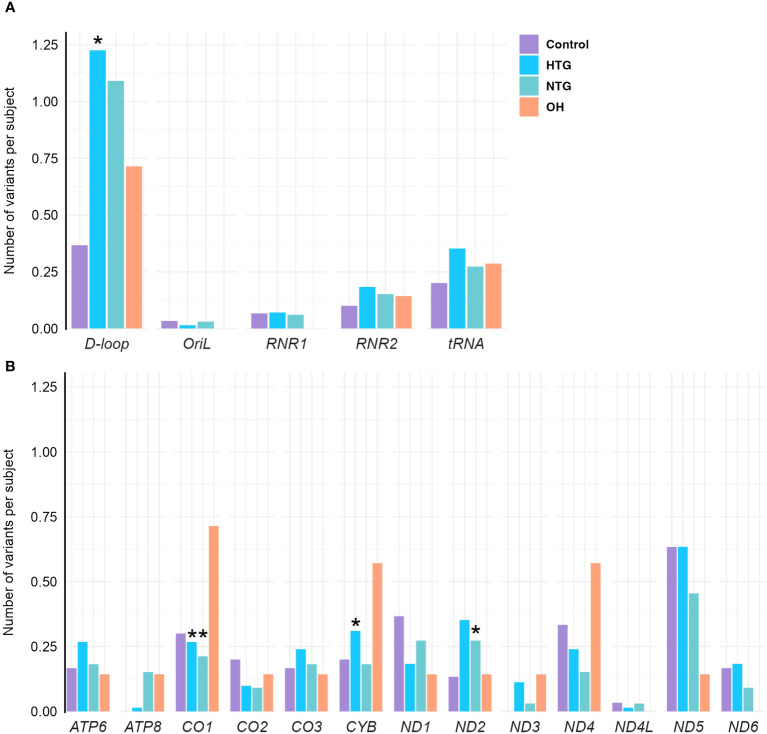
Distribution of exclusive homoplasmic variants per subject either only present in HTG, NTG, and OH groups, or in the control-group in the mtDNA **(A)** non-coding *D-loop* and *Oril* regions, *RNR1, RNR2* and *tRNA* coding regions **(B)** protein-coding regions of OXPHOS subunits *ATP6*, *ATP8*, *CO1*, *CO2*, *CO3*, *CYB*, *ND1*, *ND2*, *ND3*, *ND4*, *ND4L*, *ND5*, and *ND6*, in the Controls, HTG, NTG, and OH subjects. **(A)** A significantly higher number of exclusive variants per subject is observed in HTG patients in the *D-loop*(*) region in contrast to controls. **(B)** A significantly lower number of exclusive variants per subject is observed in the NTG and HTG groups for *CO1*(*), and a significantly higher number in *ND2*(*) for the NTG group and for *CYB*(*) in the HTG group is observed in comparison to controls for the protein-encoding mtDNA regions. Number of of variants in each subject per group and per region are fitted to a zero-inflated Poisson model. Data is displayed as the number of variants per subject. The total number of exclusive variants in each gene and group is divided by the respective size of the group. HTG, high tension glaucoma; NTG, normal tension glaucoma; OH, Ocular hypertensive.

As some variants were highly frequent in all groups, they could mask a difference in the distribution of the rarer variants. Therefore, we decided to study the distribution of homoplasmic exclusive variants in the different mtDNA regions which were either only present in HTG, NTG, and OH groups, or in the control-group. In this way, we explored if exclusive variants would appear more prominently and specifically in certain mtDNA regions for the glaucoma and OH groups. When fitting the number of exclusive variants per subject in the different areas to a zero-inflated Poisson model we observed significant relevant interactions (**Table 1**). HTG patients displayed a significantly higher number of exclusive variants per subject in the *D-loop* (~1.23 variants/subject) in comparison to controls (~0.35 variants/subject) (**Figure 3A**). Furthermore, a significantly different number of exclusive variants were observed for the *CO1* gene in both glaucoma groups, as well as for the *CYB* gene in the HTG group and *ND2* gene in the NTG group in comparison to controls (**Figure 3B**). However, when variants in these protein-coding genes were divided into synonymous and non-synonymous for the protein-coding genes, neither could explain the differences between groups (**Supplementary Figure 1**).

**Table 1:** Best fitting zero-inflated Poisson model of the number of exclusive variants per subject in the different study groups and regions of the mtDNA.

Best fitting count model coefficients (Poisson with log link):		
	Estimate	Standard error
**(Intercept)**	0.26617	0.15674
**HTG**	0.53276	0.17859
**NTG**	0.05724	0.18881
**OH**	-0.32485	0.27136
***CO1***	0.30426	0.14133
***CO3***	0.16077	0.0954
***CYB***	0.52883	0.145
***D-LOOP***	0.25728	0.05617
***ND1***	0.30555	0.07777
***ND2***	0.07948	0.099
***ND4***	0.21515	0.09996
***ND5***	0.16509	0.04723
***RNR1***	0.26264	0.13291
***RNR2***	0.35818	0.09761
***TH***	0.59223	0.26027
***TQ***	0.27559	0.22942
**HTG : *CO1***	-0.30734	0.18343
**HTG : *CYB***	-0.38691	0.1679
**HTG : *D-LOOP***	-0.09412	0.06483
**NTG : *CO1***	-0.58909	0.28105
**NTG : *ND2***	0.46107	0.34661
Zero-inflation model coefficients (Binomial with logit link):		
	Estimate	Standard error
**(Intercept)**	-5.5644	2.4926
**HTG**	-0.2436	4.3283
Log-likelihood: -241.8 on 23 Df		
**AIC: 529.6801**		

The best fitting Zero-inflated Poisson model selected by smaller Akaike information criterion (AIC). Interactions of groups with genes (:) represent a significant difference in the respective gene in contrast to controls. HTG, high tension glaucoma; NTG, normal tension glaucoma; OH, ocular hypertensive.

**Figure 4:**
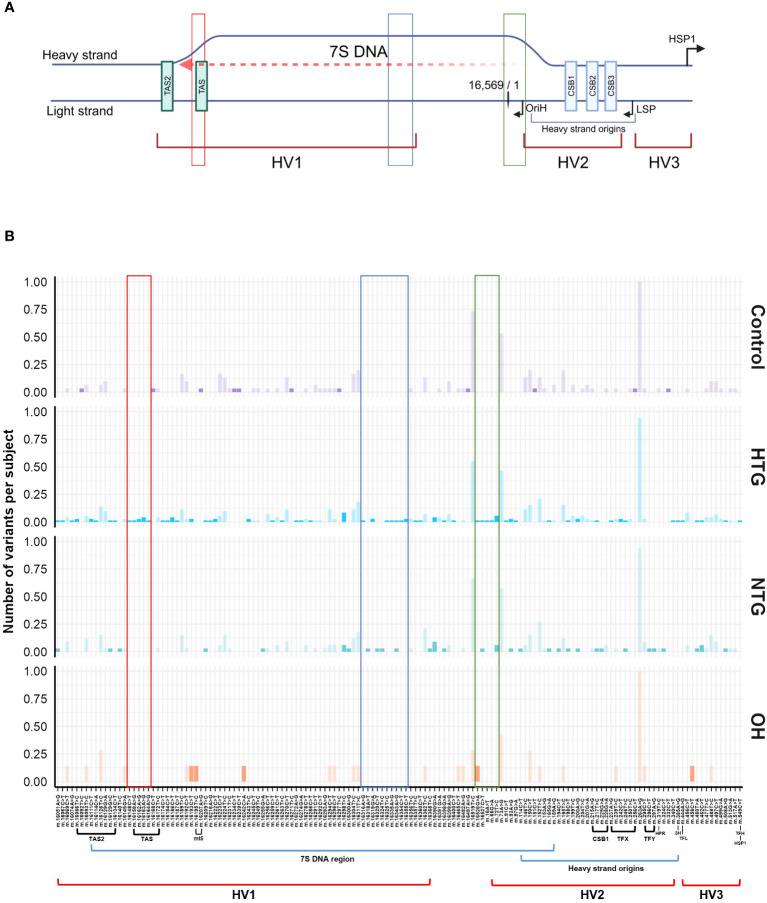
General Scheme of the major related sub-regions **(A)** and distribution of variants along the *D-loop* region **(B)**. **(A)** The different sub-areas of the *D-loop* are depicted in both the heavy and the light strands of the mtDNA [Adapted from Falkenberg (31), Nicholls and Minczuk (61)]. 3 hypervariable regions (HV1, HV2, and HV3); heavy strand origins zone and the respective origin of replication of the heavy strand (OriH); both light and the major heavy strand promoter (LSP, HSP1) and its respective direction; conserved sequence blocks 1,2 and 3 (CSB1,2,3) and termination associated sequences (TAS, TAS2). Binding sites for the transcription factor mtTF1 are situated from between CSB1 and CSB2 till almost the HSP. The 7S DNA area, where the *D-loop* triple stranded structure is formed by the addition of a short 7S DNA strand, is depicted by a red dashed arrow. Created with BioRender.com **(B)** Patient (HTG, NTG) and OH exclusive variants and control exclusive variants are displayed in a bright color, while non-exclusive variants are displayed in a faint color. Areas of the region that only present variants in the HTG and/or NTG groups are highlighted in red, blue and green boxes. The sub-regions inside the *D-loop* are shown below the x-axis, only if a variant was present in the respective sub-region. Data is displayed as the number of variants per subject The total number of subjects presenting each variant and group is divided by the respective size of the group. MT5, control element; HPR, replication primer; HTG, high tension glaucoma; NTG, normal tension glaucoma; OH, Ocular hypertensive; TFX, TFY, TFH, TFL, mtTF1 binding sites; 3H, mt3 heavy-strand control element.

The significant interaction between HTG and the higher number of exclusive variants per subject in the *D-loop* region prompted us to study if certain variants were driving this result. No specific exclusive variant was responsible for this difference, but several exclusive variants were present in 1 up to 6 HTG patients (**Supplementary Table 1**). Next, we divided the *D-loop* variants among the different *D-loop* sub-regions (60) (**Figure 4A**). In the 7S DNA sub-region within the Hypervariable region 1 (HV1) we found that 42% of the HTG and 27% of the NTG subjects presented variants in this area, whereas this was approximately 14% in the case of Controls and OH. In this sub-region, variants between positions 16,316 and 16,355 were only found in HTG and NTG patients (**Figure 4B**, highlighted in blue). We also observed that exclusive variants between positions 16,148 to 16,164, which are near and inside the termination-associated sequence (TAS), were only present in HTG patients (**Figure 4B**, highlighted in red). Additionally, between positions 16,526 and 72, inside the 7S DNA sub-region within the hypervariable region 2 (HV2), only HTG and NTG patients presented exclusive variants, with the exception of variant m.16526G>A that was also present in a OH subject (**Figure 4B**, highlighted in green). The most frequent exclusive variants, which were found in ≥3 patients in at least one of the glaucoma groups, were observed in the previously mentioned regions e.g., m.16186C>T (3 HTG patients) and m.16298T>C (6 HTG and 2 NTG subjects) in the 7S DNA sub-region within HV1, but also m.72T>C (4 HTG and 2 NTG patients) in 7S DNA sub-region inside HV2, m.16163 A>G (3 HTG patients) inside the TAS and m.16390G>A (3 HTG and 3 NTG patients) inside the 7S DNA sub-region (**Figure 4B**; **Supplementary Table 1**). We also observed that 38% of the HTG and 42% of NTG did not have any exclusive variants in the *D-loop* region.

For all subjects, we have previously studied the number of mtDNA copies in the same buffy coat DNA samples (43). We found that HTG subjects presented a significantly lower mtDNA copy number than controls and NTG subjects. However, no correlation was observed between the number of exclusive *D-loop* variants and mtDNA copy number nor specifically for the HTG group. Also, patients without any *D-loop* variant could present low mtDNA copy number, meaning lower than control group median. In addition, we studied the relation between the most frequent and patient exclusive *D-loop* variants (m.72T>C, m.16163 A>G, m.16186C>T, m.16298T>C, m.16390G>A) and the mtDNA copy number in glaucoma patients. While the majority of glaucoma patients presenting these variants had a mtDNA copy number below the controls median, no significant relation between these variants and mtDNA copy number of glaucoma patients was found.

## 4 Discussion

In this study, we investigated if mtDNA variants and their distribution in different mtDNA regions could be risk factors for developing glaucoma. As no differences were found between the groups in any of the non-coding or coding regions, we decided to study the distribution of exclusive variants in the different mtDNA regions which were either only present in HTG, NTG, and OH groups, or in the control-group. We observed that subjects with POAG, more specifically HTG, have a significantly higher number of exclusive homoplasmic variants per subject in the *D-loop* region, with a higher percentage of those being in the 7S DNA within the HV1 region. At the same time, we also observed that a subgroup of HTG subjects did not present any exclusive variant in the *D-loop* region.

The *D-loop*, covering the whole non-coding mtDNA region (NC_012920.1), is a special structure, composed of 3 different hypervariable regions (HV1, 2, 3), the 7S DNA region, and other sub-regions (60) involved in mtDNA replication and transcription and stability and formation of the *D-loop*. These include the light and heavy strand promoters, the binding sites for the transcription factor mtTF1, conserved sequence blocks (CSB1,2,3), termination-associated sequences (TAS, TAS2) and others (31, 61–63) (**Figure 4A**). How the *D-loop* and its related sub-regions exactly regulate mtDNA replication and transcription together with the nuclear DNA encoded proteins is largely unknown, but previous studies have shown that *D-loop* variants can impact the mtDNA replication in a positive and negative way (64–67). Therefore, we investigated a possible correlation between the number of exclusive *D-loop* variants per subject and the low mtDNA copy number in HTG patients (43). No overall correlation nor specific study group association was observed. In addition, while we noticed that most glaucoma patients presenting the most frequent and patient exclusive variants (m.72T>C, m.16163 A>G, m.16186C>T, m.16298T>C, m.16390G>A) had a lower mtDNA copy number than the median of the controls, there was no statistically significant association observed. Whether a reduced mtDNA copy number due to alterations in the *D-loop* might be a risk factor in a subgroup of glaucoma subjects remains uncertain. Larger study cohorts, in which control region variants and mtDNA copy number can be correlated, should be able to clarify a possible functional role for each of the variants observed. Additionally, we have also found that a subgroup of HTG subjects existed with a low mtDNA copy number that did not present any exclusive variant in the *D-loop* region. This suggested that a reduced mtDNA copy number can have multiple causes, like variants in nuclear genes involved in mtDNA replication, environmental factors or accelerated aging (68, 69). Previous research showed correlation between a higher number of exclusive *D-loop* variants and a decrease in mtDNA copy number in blood and tumoral tissues (70–73). However, blood and tumoral tissue mtDNA without *D-loop* variants were also reported presenting a lower number of mtDNA copies, as observed in our data (70, 71).

Two of the most frequently observed patient-exclusive variants were mentioned in previous POAG and mtDNA replication related literature. The variant m.16390G>A was found significantly enriched in a large African-American POAG cohort (74). Other studies have found this variant in a higher frequency in Tunisian patients with type 2 diabetes (75) or related to different pathologies such as non-small cell lung cancer. In the latter, the possible negative effect on mtDNA replication was emphasized (76). The m.16390G>A variant is found downstream of the heavy strand origins, at the 7S DNA triple strand zone in the direction of the replication fork formation for the unidirectional formation of the leading/heavy strand (64, 77). The variant m.16163A>G in the TAS region has been found before in a study of POAG subjects and controls of European descent (78). In our study, only HTG patients presented this variant, who simultaneously harbored m.16186C>T. Variants near (m.16148C>T) and within positions 16,148 and 16,164 in the TAS region are only found in HTG patients. The TAS and CSB regions have been suggested to be functionally important in both transcription, replication and *D-loop* stability, and variants in these areas have been proposed to affect these processes. However, the mechanism behind it is largely unknown (65, 66, 79–81). Further study on the potential mechanism by which variants in the TAS region or m.16390G>A in the 7S DNA region might cause a disadvantageous mtDNA replication could be done in *in vitro* cell models.

The identification of *D-loop* variants that lead to a reduced mtDNA copy number is important to characterize the glaucoma subgroup that can benefit from treatments aimed at increasing mitochondrial biogenesis. This treatment has been previously proposed for optic neuropathies to increase the RGCs viability and our data might help identify the subgroup of patients for which this treatment will be beneficial (82). In LHON, a primary mitochondrial disease caused by homoplasmic mtDNA mutations and characterized by optic nerve neurodegeneration, a systemic increase in mtDNA copies has been observed in asymptomatic carriers in contrast to controls and affected carriers (83–86). This seems a comparable compensatory mechanism. But the data on *D-loop* variants in relation to mtDNA copy number has impact beyond the optic neuropathies. As indicated above, a reduced mtDNA copy number might be a risk factor in common disease, like type 2 diabetes or in certain types of cancer (87). Also in mitochondrial replacement therapy, replication differences between donor and recipient mtDNA might have serious consequences for the eventual outcome of the treatment (65, 88–92). Furthermore, the process of mtDNA replication and transcription is not yet fully understood and the relevant regions in the *D-loop* have not been properly identified, so our data can also contribute to select the variants, which might have a functional role in this complex process.

In conclusion, variants in the *D-loop* region may be a possible risk factor for a glaucoma subgroup, possibly by affecting mtDNA replication leading to lower systemic mtDNA copy number. Further studies need to confirm and resolve how big this subpopulation of patients is. Impaired replication leading to lower mtDNA copy number, in consonance with glaucoma risk factors such as IOP and aging, could compromise mitochondrial function and increase the vulnerability of the post-mitotic and high energy required RGCs (16, 19) in a subgroup of glaucoma subjects.

## Data availability statement

The datasets presented in this article are not readily available because of privacy concerns. The data will be made available upon request and consideration. Requests to access the datasets should be directed to a.vallbonagarcia@maastrichtuniversity.nl and f.vanden.biggelaar@mumc.nl.

## Ethics statement

The studies involving humans were approved by Medical ethics review committee of the azM and Maastricht University (METC azM/UM). The studies were conducted in accordance with the local legislation and institutional requirements. The participants provided their written informed consent to participate in this study.

## Author contributions

AV-G: Conceptualization, Data curation, Formal analysis, Investigation, Methodology, Project administration, Software, Writing – original draft, Writing – review & editing, Visualization. PL: Data curation, Formal analysis, Software, Writing – review & editing, Conceptualization, Investigation, Visualization. RK: Methodology, Project administration, Writing – review & editing. AS: Formal analysis, Methodology, Software, Writing – review & editing. NN: Data curation, Formal analysis, Software, Writing – review & editing. FvT: Data curation, Formal analysis, Investigation, Writing – review & editing. IH: Methodology, Project administration, Writing – review & editing. RH: Methodology, Project administration, Writing – review & editing. MvG: Conceptualization, Investigation, Methodology, Project administration, Resources, Writing – review & editing. BB: Investigation, Project administration, Writing – review & editing. IdC: Writing – review & editing. CW: Conceptualization, Funding acquisition, Investigation, Project administration, Resources, Supervision, Writing – review & editing. TG: Conceptualization, Funding acquisition, Investigation, Methodology, Project administration, Resources, Supervision, Visualization, Writing – original draft, Writing – review & editing. HS: Conceptualization, Investigation, Project administration, Resources, Supervision, Visualization, Writing – original draft, Writing – review & editing, Formal analysis, Methodology.
