# Supplementary material for: Mitochondrial DNA D-loop variants correlate with a primary open-angle glaucoma subgroup
Source: Front Ophthalmol (Lausanne). 2024 Jan 17;3:1309836. doi: 10.3389/fopht.2023.1309836 (PMC11182222; doi:10.3389/fopht.2023.1309836)
Supplement: Supplementary file 1 [file DataSheet_1.pdf]

Supplement

Mitochondrial DNA *D-loop* variants correlate with a primary open-angle glaucoma subgroup

Antoni Vallbona-Garcia[A,B,C,G], Patrick J. Lindsey[B], Rick Kamps[B], Alphons P.M. Stassen[D], Nhan Nguyen[B], Florence H.J. van Tienen[B,C], Ilse H.J. Hamers[B], Rianne Hardij[B], Marike W van Gisbergen[E,F], Birke J. Benedikter[A,C], Irenaeus F.M. de Coo[B], Carroll A.B. Webers[A,C], Theo G.M.F Gorgels[A,C,*], Hubert J.M. Smeets[B,C,*].

A University Eye Clinic Maastricht, Maastricht University Medical Center+, Maastricht, The Netherlands.

B Department of Toxicogenomics, Maastricht University, Maastricht, The Netherlands

C School for Mental Health and Neuroscience, Maastricht University, Maastricht, The Netherlands

D Department of Clinical Genetics, Maastricht University Medical Center, Maastricht, The Netherlands

E Department of Dermatology, Maastricht University Medical Center, Maastricht, The Netherlands

F GROW School for Oncology and Reproduction, Maastricht University, the Netherlands

G Corresponding author: Antoni Vallbona Garcia, a.vallbonagarcia@maastrichtuniversity.nl

*** These authors contributed equally to the work**

Supplementary Figures

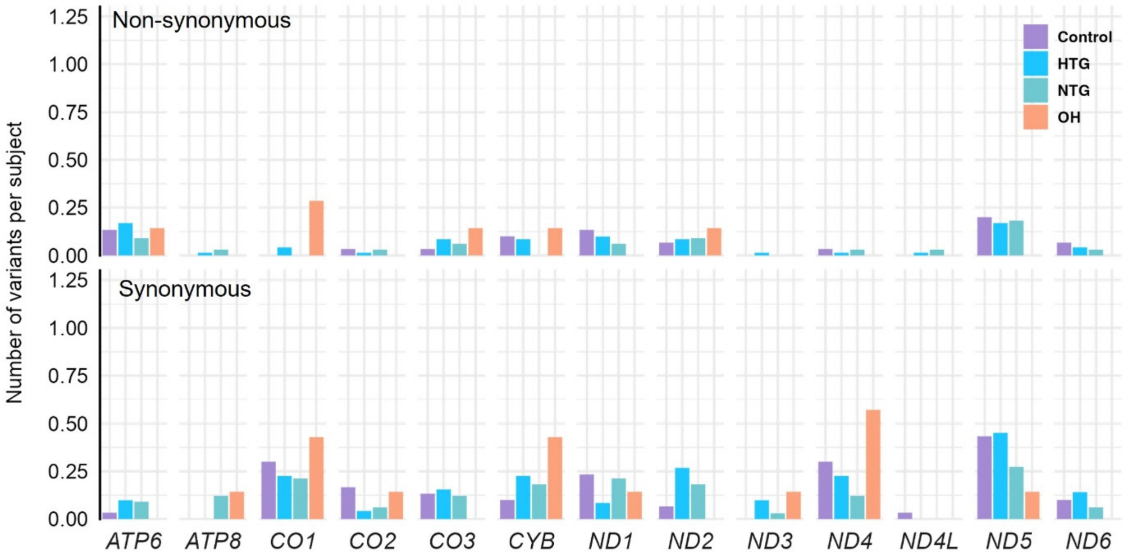

Supplementary figure 1. Distribution of synonymous and non-synonymous exclusive homoplasmic variants per subject either only present in HTG, NTG, and OH groups, or in the control group in the OXPHOS subunits protein-coding genes *ATP6*, *ATP8*, *CO1*, *CO2*, *CO3*, *CYB, ND1, ND2, ND3, ND4, ND4L, ND5,* and *ND6*, in the Controls, HTG, NTG, and OH subjects. No differences between groups are observed when the number of synonymous and non-synonymous variants in each subject per protein-encoding gene are fitted to a zero-inflated Poisson model. Data is displayed as the number of variants per subject. The total number of exclusive variants in each gene and group is divided by the respective size of the group. HTG: high tension glaucoma; NTG: normal tension glaucoma; OH: Ocular hypertensive.

Supplementary tables

Supplementary Table 1: Number of subjects in each group with specific *D-loop* exclusive variants.

VARIANT	CONTROL	HTG	NTG	OH
**m.16A>T**	0	1	0	0
**m.66G>A**	0	1	0	0
**m.72T>C**	0	4	2	0
**m.91C>T**	0	1	0	0
**m.93A>G**	0	2	1	0
**m.97G>A**	0	1	0	0
**m.114C>T**	0	0	1	0
**m.151C>T**	1	0	0	0
**m.185G>A**	0	1	1	0
**m.194C>T**	0	0	1	0
**m.198C>T**	0	1	1	0
**m.200A>G**	0	2	1	0
**m.217T>C**	0	1	1	0
**m.225G>A**	0	0	1	0
**m.237A>G**	0	1	0	0
**m.242C>T**	0	2	0	0
**m.246T>C**	0	1	1	0
**m.256C>T**	1	0	0	0
**m.296C>T**	0	0	1	0
**m.297A>G**	0	0	1	0
**m.324C>T**	0	0	1	0
**m.332C>T**	1	0	0	0
**m.340C>T**	0	1	0	0
**m.385A>G**	0	1	0	0
**m.444A>G**	0	1	0	0
**m.458C>T**	0	0	0	1
**m.460T>A**	0	1	1	0
**m.462C>T**	0	1	2	0
**m.508A>G**	0	1	1	0
**m.549C>T**	0	1	0	0
**m.16051A>G**	0	1	1	0
**m.16067C>T**	0	1	0	0
**m.16074A>G**	0	1	0	0
**m.16086T>C**	0	2	0	0
**m.16092T>C**	1	0	0	0
**m.16111C>T**	0	2	0	0
**m.16114C>A**	0	1	0	0
**m.16129G>C**	0	1	1	0
**m.16134C>T**	0	1	0	0
**m.16140T>C**	0	0	1	0
**m.16148C>T**	0	1	0	0

**m.16158A>G**	0	1	0	0
**m.16162A>G**	0	2	0	0
**m.16163A>G**	0	3	0	0
**m.16164A>G**	0	1	0	0
**m.16168C>T**	1	0	0	0
**m.16174C>T**	0	1	0	0
**m.16184C>T**	0	1	0	0
**m.16186C>T**	0	3	0	0
**m.16187C>T**	0	1	0	0
**m.16193C>T**	0	2	0	1
**m.16195T>C**	0	0	0	1
**m.16207A>G**	1	0	0	0
**m.16209T>C**	0	1	1	0
**m.16216A>G**	0	1	0	0
**m.16222C>T**	0	2	0	0
**m.16234C>T**	1	0	0	0
**m.16239C>T**	1	0	0	0
**m.16242C>A**	0	0	0	1
**m.16243T>C**	0	2	0	0
**m.16255G>A**	0	0	1	0
**m.16259C>T**	0	1	0	0
**m.16263T>C**	0	1	0	0
**m.16271T>C**	1	0	0	0
**m.16272A>G**	0	1	0	0
**m.16274G>A**	0	1	0	0
**m.16286C>T**	0	1	0	0
**m.16297T>C**	1	0	0	0
**m.16298T>C**	0	6	2	0
**m.16300A>G**	0	0	1	0
**m.16316A>G**	0	1	0	0
**m.16318A>T**	0	0	1	0
**m.16319G>A**	0	2	0	0
**m.16320C>T**	0	0	1	0
**m.16324T>C**	0	0	1	0
**m.16325T>C**	0	1	0	0
**m.16335A>G**	0	1	0	0
**m.16343A>G**	0	1	1	0
**m.16354C>T**	0	1	0	0
**m.16355C>T**	0	2	0	0
**m.16357T>C**	0	1	0	0
**m.16360C>T**	0	1	0	0
**m.16368T>C**	0	0	1	0
**m.16390G>A**	0	3	3	0
**m.16398G>A**	0	1	1	0
**m.16400C>T**	0	1	0	0
**m.16497A>G**	1	0	0	0

**m.16526G>A**	0	1	0	1
**m.16527C>T**	0	1	1	0

HTG: high tension glaucoma; NTG: normal tension glaucoma; OH: ocular hypertensive.
